# Making Sense of Optogenetics

**DOI:** 10.1093/ijnp/pyv079

**Published:** 2015-07-25

**Authors:** Akash Guru, Ryan J Post, Yi-Yun Ho, Melissa R Warden

**Affiliations:** Department of Neurobiology and Behavior, Cornell University, Ithaca, NY (Mr Guru and Post, Ms Ho, and Dr Warden).

**Keywords:** channelrhodopsin-2, ChR2, halorhodopsin, optogenetics, OptoXR, NpHR

## Abstract

This review, one of a series of articles, tries to make sense of optogenetics, a recently developed technology that can be used to control the activity of genetically-defined neurons with light. Cells are first genetically engineered to express a light-sensitive opsin, which is typically an ion channel, pump, or G protein–coupled receptor. When engineered cells are then illuminated with light of the correct frequency, opsin-bound retinal undergoes a conformational change that leads to channel opening or pump activation, cell depolarization or hyperpolarization, and neural activation or silencing. Since the advent of optogenetics, many different opsin variants have been discovered or engineered, and it is now possible to stimulate or inhibit neuronal activity or intracellular signaling pathways on fast or slow timescales with a variety of different wavelengths of light. Optogenetics has been successfully employed to enhance our understanding of the neural circuit dysfunction underlying mood disorders, addiction, and Parkinson’s disease, and has enabled us to achieve a better understanding of the neural circuits mediating normal behavior. It has revolutionized the field of neuroscience, and has enabled a new generation of experiments that probe the causal roles of specific neural circuit components.

## Introduction

Progress towards understanding the neural circuits of the brain has historically relied on the development of new technologies that advance our ability to observe and control neuronal activity. These techniques have allowed us to dissect the detailed workings of the neural circuits underlying natural behavior, and they have also enabled us to understand some facets of how neural circuits become dysfunctional in disease states. Some model organisms (worm, sea slug, lobster, and crab, among others) have the significant advantage that their relatively small nervous systems permit experimental perturbation of individually identifiable single neurons, an approach that has given rise to a sophisticated understanding of the functional wiring diagram controlling behavior in these organisms. Vertebrate animals (fish, mice, rats, birds, and primates), on the other hand, have a much larger, more complex, and highly variable nervous system that is not tractable using this kind of approach. The vertebrate brain, encompassing hundreds of millions of neurons in rodents and hundreds of billions of neurons in humans, contains many different cell types with distinct molecular expression patterns, physiological activity, and topological connectivity, which are intermingled in a highly heterogeneous network. Although substantial progress has been made towards understanding vertebrate neural circuits, there are significant limitations associated with the techniques classically used to probe and control brain function. While powerful, intracranial lesions and electrical stimulation affect spatially defined brain regions without restricting their action to a particular kind of neuron, and cell type–specific pharmacology and transgenic or viral manipulation of gene expression have relatively low temporal resolution.

Optogenetics has ushered in a new era of potent and targeted control over multiple aspects of neural function. Genetic and optical methods applied together allow tight spatial and temporal control of the activity of specific kinds of neurons in the living brain, a revolutionary advance that will allow us to achieve an unprecedented understanding of neural circuit function in behaving animals. In brief, neurons are first genetically engineered (using a variety of mechanisms, described later) to express light-sensitive proteins (opsins). When these neurons are then illuminated with light of the correct frequency they will be transiently activated or inhibited or their signaling pathways will be modulated, depending on the particular kind of opsin that was chosen for expression. Cell type–specific expression is typically achieved with transgenic animals, viral vectors, or a combination, and spatially restricted light application allows for further refinement in targeting to specific brain regions. Light can be applied in a variety of temporal patterns in order to optimally influence neuronal function (permitting experimental control of spike frequency and burstiness, among other parameters), and may be restricted to specific short behavioral epochs. Optogenetic tools, first used to control neuronal function a decade ago (Boyden et al., 2005), have been extensively developed over the intervening years and now include a vast array of proteins that allow control of neural activity over a range of timescales, control of biochemical activity within the cell, control of multiple neural channels in parallel, and, most recently, control of neural activity in parallel with optical monitoring of neural activity.

### What Are Optogenetic Actuators?

**Figure 1.**
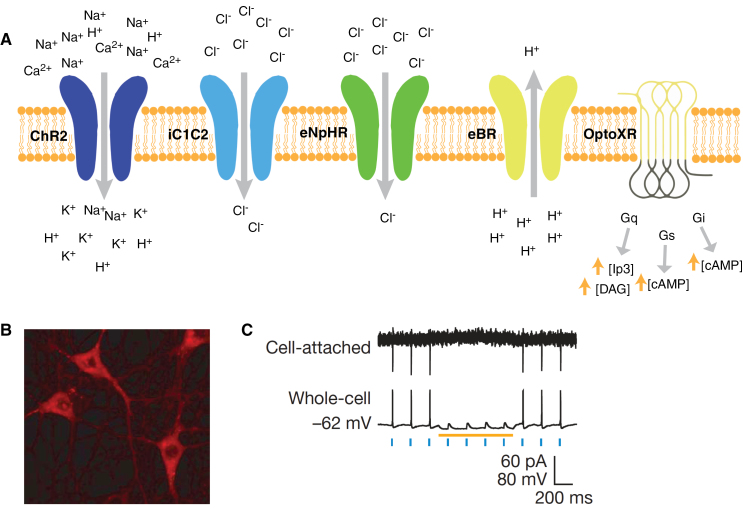
(A) Opsins are membrane-bound proteins that are activated with light, which results in cell activation (depolarization), inhibition (hyperpolarization), or modulation of intracellular signaling cascades. A wide variety of opsins are now available. Illustrated here are ChR2 (a cation channel used to stimulate neural activity), iC1C2 (a newly developed chloride channel used to inhibit neural activity), eNpHR3.0 (a chloride pump used to inhibit neural activity), eBR (a proton pump used to inhibit neural activity), and OptoXR (a G protein–coupled receptor used to modulate intracellular signaling cascades). (B) Neurons in culture expressing a ChR2-mCherry fusion protein. (C) Cell-attached and whole-cell recordings from a neuron expressing both ChR2 and NpHR. Note that individual spikes can be elicited with a short pulse of blue light (which activates ChR2) and that these spikes can be blocked with continuous yellow light (which activates NpHR). Panel A adapted with permission from Fenno et al., 2011, and panels B and C adapted with permission from Zhang et al., 2007.

Optogenetic actuators are proteins that modify the activity of the cell in which they are expressed when that cell is exposed to light (Figure 1). These actuators can be used to induce single or multiple action potentials (which can be organized into regular spike trains or which can be pseudo-random at a user-controlled rate), suppress neural activity, or modify biochemical signaling pathways, with millisecond control over the timing of events. The most powerful and widely used actuators are opsins—naturally occurring light-sensitive transmembrane proteins—that are found in a variety of organisms ranging from microbes to primates, and that can be used as found in nature or engineered to optimize functioning. Naturally occurring opsins can be broadly categorized into two major classes: microbial opsins (Type I) and vertebrate opsins (Type II). Type I opsins are found in prokaryotic and eukaryotic microbial organisms, including bacteria, archaea, and algae, and are composed of a single membrane–bound protein component that functions as a pump or channel (Oesterhelt and Stoeckenius, 1973; Nagel et al., 2002, 2003). These opsins are used by their host microorganisms for a variety of functions, including navigation towards sources of energy and away from hazardous environments, and control the intracellular concentrations of a variety of ions and the beating of flagella. Type II opsins are found in animal cells and are primarily used for vision and modulating circadian rhythms. These opsins are G protein–coupled receptors, initiate a signaling cascade upon activation, and consequently produce slower changes in neural activity than Type I opsins. Type I opsins were used in the first optogenetics experiments to control neuronal function, both because of the ease of genetic engineering using a single component protein and because of their faster kinetics, and remain the primary (but not exclusive) source for new natural and engineered opsins.

### How Do Optogenetic Actuators Work?

Opsins of both types require retinal, a form of vitamin A that isomerizes upon absorption of a photon, in order to function. When retinal binds to the opsin the retinal-opsin complex becomes light sensitive, and if a photon strikes the retinal in this state its resulting photoisomerization will induce a conformational change in the opsin. This leads to channel opening or pump activation, a change in membrane potential, and ultimately the activation or inhibition of neuronal activity. Therefore, retinal must be present in order for optogenetic actuators to function. Fortunately, particularly for the early proof-of-principle experiments, retinal is already present in sufficient quantities in mammalian neural tissue to permit the use of optogenetic tools without exogenous retinal supplementation. However, invertebrate model systems such as *Drosophila* do need retinal supplementation through their diet in order for optogenetic effectors to function. Here we review the different classes of optogenetic actuators, grouped by their effect on neural activity or signaling.

### Optogenetic Stimulation of Neural Activity

#### Channelrhodopsins

Channelrhodopsins (ChRs) are light-gated ion channels discovered in *Chlamydomonas reinhardtii,* a unicellular green alga (Nagel et al., 2002, 2003, 2005b). The first use of a microbial opsin to control the spiking activity of neurons utilized Channelrhodopsin-2 (ChR2), one of two channelrhodopsins expressed by this organism (Boyden et al., 2005). ChR2 is a light-gated nonspecific cation channel which, when illuminated with blue light, opens and allows the passage of cations and the subsequent depolarization of the cell (Nagel et al., 2003, 2005b). In 2005 ChR2 was introduced into cultured hippocampal neurons and successfully used to control spiking activity with fine temporal precision (Boyden et al., 2005). As demonstrated by this pioneering paper, very brief (millisecond) pulses of blue light can be used to induce single action potentials in ChR2-expressing neurons, and spiking activity driven by the activation of this opsin can be controlled with high precision at frequencies approaching 30 spikes per second. This initial demonstration of the usefulness of ChR2 for the control of neural activity was soon followed by a number of reports confirming its function in neurons (Li et al., 2005; Ishizuka et al., 2006) and usefulness for addressing basic questions in neurobiology and behavior (Nagel et al., 2005a; Bi et al., 2006; Schroll et al., 2006). ChR2 has subsequently been engineered to optimize expression and photocurrent in mammalian systems (Nagel et al., 2005a; Gradinaru et al., 2007).

**Figure 2.**
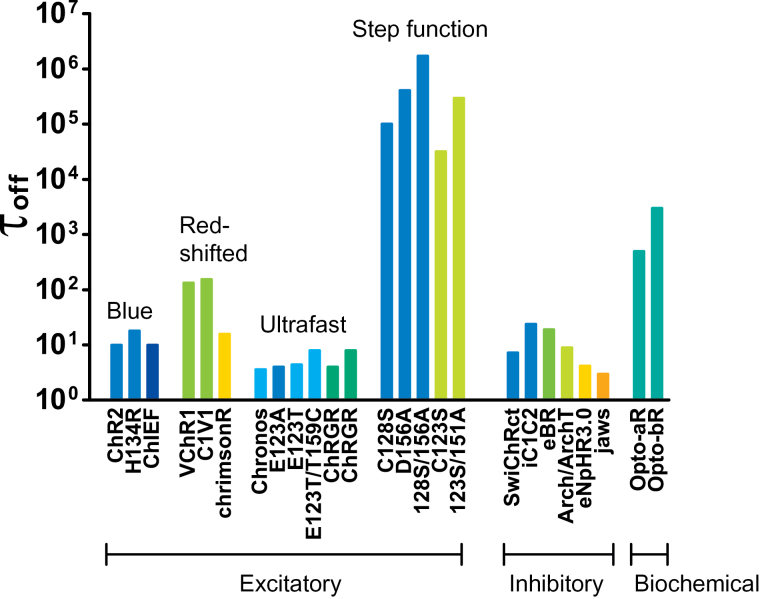
An illustration of some of the currently available optogenetic actuators. Color indicates the optimal frequency of light used for illumination, and τ_off_ indicates speed of deactivation (fast opsins have a small τ_off_ and slow opsins have a large τ_off_). Different opsins are suitable for different purposes, discussed in the text. Excitatory and inhibitory opsins are available, as are opsins that can modulate intracellular signaling cascades. Adapted from Fenno et al. 2011, with permission.

Since these initial reports the optogenetic toolbox has greatly expanded, and many different opsins with a variety of spectral, temporal, and conductive properties have been discovered or engineered and reviewed (Figure 2; Fenno et al., 2011; Yizhar et al., 2011a; Mattis et al., 2012). Microbial organisms have evolved an array of opsins that possess a diversity of functional properties, which can be used as tools useful for a range of different applications with minimal optimization. Additionally, protein engineering by targeted mutation and the creation of chimeras has been used to create a vast set of new functions, to optimize existing function, and to control the cellular targeting of opsins.

#### Ultrafast Opsins 

An area of particular interest in channelrhodopsin development has been the generation of opsins with faster temporal kinetics, achieved by accelerating opsin deactivation (off-kinetics) through targeted mutation or the creation of chimeras. Through these efforts, opsins such as ChETA and ChEF/ChIEF were developed, among others (Lin et al., 2009; Gunaydin et al., 2010; Mattis et al., 2012). These tools are suited for applications in which extremely fast temporal control of neural activity is desired at high neural firing rates (e.g. to control the activity of fast-spiking inhibitory parvalbumin neurons). In addition, these opsins reduce the occurrence of doublet or triplet spikes resulting from a single light pulse, sometimes problematic when using ChR2 if the expression level is not tightly controlled.

#### Step-Function Opsins

In some experimental paradigms it may be more desirable to modify the spontaneous firing rate of a neural population rather than control the generation of every action potential. This approach may be particularly useful in situations where more naturalistic, desynchronized spiking patterns are preferred. Step function or bi-stable opsins (SFOs) are useful tools for achieving this purpose, and were created by modifying ChR2 to stabilize the open conducting state. The first SFO was generated by introducing a point mutation of ChR2 at the C128 position [ChR2(C128A), ChR2(C128S), or ChR2(C128T)]. This mutation extends the lifetime of the channel open state to tens of seconds, thus creating a depolarizing step upon brief light illumination (Berndt et al., 2009). Another variant, ChR2/D156A, has a deactivation timescale on the order of minutes (Bamann et al., 2010). Combining these two mutations resulted in a stabilized SFO—ChR2(C128S/D156A)—that has a spontaneous deactivation lifetime of almost half an hour (Yizhar et al., 2011b). When using these opsins, photocurrents can be initiated with a brief, blue light pulse and terminated with a yellow light pulse, offering millisecond scale temporal precision of depolarization onset and offset coupled with a higher-rate spontaneous spiking pattern. Stabilized SFO is particularly useful for manipulating cellular activity in behavioral paradigms where connection to a fiber optic tether would be awkward. Because of its very long deactivation kinetics, animals may be briefly attached to a fiber optic cable for the initiation of neural activity with a blue light pulse, detached from the tether for behavioral testing, then attached again for termination of elevated neural activity with a yellow light pulse. One caveat that must be kept in mind when using SFOs is their extreme sensitivity to light (Tye and Deisseroth, 2012), which makes it possible to stimulate SFO-expressing neurons with a light source several millimeters away from infected tissue. Although this is an advantage for some applications, particularly non-invasive stimulation from outside the skull, it may be a disadvantage when attempting to use this opsin for projection-specific modulation of neural activity with axonal illumination (discussed below).

#### Spectrally Shifted Excitatory Opsins

Effort has focused on the development of opsins with shifted excitation spectra in order to achieve independent optical control of different populations of neurons. The development of red-shifted opsins has been a primary initial objective, for two principal reasons. First, such an opsin could be used in combination with blue light–sensitive ChR2 with minimal spectral overlap. Second, a long, wavelength-sensitive opsin would be useful in its own right to enable deep penetration of light into tissue with reduced scattering, which may be useful for non-invasive light delivery. The first red-shifted opsin, VChR1, was identified in *Volvox carteri* and had an excitation maximum at 535nm, significantly red-shifted when compared with ChR2 at 460nm (Zhang et al., 2008). Although utilization of VChR1 was hindered by low photocurrent in mammalian systems, C1V1 (a variant generated by fusing the N-terminal sequence of ChR1 with the C-terminal sequence of VChR1) also exhibits red-shifted peak absorption at 539nm and has seen widespread adoption (Yizhar et al., 2011b), though it should be noted that this variant has relatively long deactivation kinetics compared to ChR2. Another variant, red-activatable channelrhodopsin (ReaChR), has enhanced membrane trafficking and expression in mammalian cells with faster kinetics and higher photocurrents. It is also significantly more red-shifted, with peak response at 590–630nm, and has been used to drive neuronal spiking through the intact skull (Lin et al., 2013).

Although the opsins described above show peak excitation for red-shifted wavelengths of light, they also exhibit some residual absorption of blue light. This can result in a degree of cross-talk between channels unless care is taken to precisely calibrate the expression level and light power. A newly-discovered pair of opsins, however, provides a significant advance towards the resolution of this problem (Klapoetke et al., 2014). These optical tools, discovered through *de novo* sequencing of opsins from over 100 algal species, are Chrimson (an opsin with an excitation spectrum 45nm red-shifted from previous channelrhodopsins) and Chronos (a blue light– and green light–sensitive opsin with high light sensitivity and fast kinetics). When these opsins are used in combination, the low light power required to activate Chronos doesn’t elicit a significant response in Chrimson, and the red-shifted light used to activate Chrimson doesn’t elicit a response in Chronos. This combination offers two-color activation of neuronal spiking in independent neuronal populations without detectable crosstalk in mouse brain slices. Further studies are required to validate the application of these tools in freely behaving animals.

### Optogenetic Inhibition of Neuronal Activity

#### Chloride Pumps

Inhibition of neuronal activity is critical for probing the computational roles of neural circuits, and can complement excitatory tools by allowing investigators to test the necessity of individual circuit components. One of the most efficient and widely used inhibitory opsins, NpHR, is a halorhodopsin from the archaeon *Natronomonas pharaonis* (Han and Boyden, 2007; Zhang et al., 2007). NpHR pumps chloride ions into the cell upon light activation, resulting in hyperpolarization. Although the initial mammalian codon-optimized form of this opsin did not traffic well to the cell membrane and accumulated in the endoplasmic reticulum, subsequent engineering led to a series of revisions culminating in eNpHR3.0, an opsin with improved surface membrane localization and a large photocurrent (Gradinaru et al., 2008, 2010; Zhao et al., 2008). With an excitation maximum at 590nm, eNpHR3.0 can be driven by green, yellow, or red wavelengths of light, enabling the use of less expensive laser systems.

#### Proton Pumps

Proton pumps can also be used to inhibit neurons through hyperpolarization, by pumping protons out of the cell, and have some features that make them desirable alternatives to chloride pumps, which include fast recovery from inactivation and high light-driven currents. Arch (archaerhodopsin-3 from *Halorubrum sodomense*), Mac (from the fungus *Leptosphaeria maculans*), ArchT (an archaerhodopsin from *Halorubrum* strain TP009), and eBR (an enhanced version of bacteriorhodopsin from *Halobacterium salinarum*) are proton pumps that show robust efficiency in inhibition (Chow et al., 2010; Gradinaru et al., 2010; Han et al., 2011). Initial variants of these opsins, like NpHR, were plagued by issues with cellular localization and toxicity, but enhanced versions that have eliminated or greatly reduced these problems have since been developed (Mattis et al., 2012). Recent work has demonstrated that inhibition of eNpHR3.0-expressing neurons may render the inhibited neuron transiently more excitable due to a chloride-driven shift in the type-A γ-aminobutyric acid (GABA_A_) receptor reversal potential (Ferenczi and Deisseroth, 2012; Raimondo et al., 2012), which may point towards a proton pump inhibitor as the opsin of choice for some experiments.

#### Spectrally Shifted Inhibitory Opsins

More recently, spectrally shifted inhibitory opsins have been developed. The chloride and proton pump inhibitory opsins, discussed above, are significantly red-shifted from ChR2: eNpHR3.0 has an excitation maximum at 590nm, while eArch3.0, eArchT3.0, and eMac3.0 have maxima between 520 and 550nm. Development has focused both on finding or developing even more red-shifted inhibitory opsins, for maximal and potentially non-invasive light penetration into the brain, and on blue-shifted inhibitory opsins, for multichannel control with currently available inhibitory opsins. Recently, there has been significant progress towards the development of highly red-shifted inhibition. Chuong et al. (2014) discovered and optimized a red-shifted cruxhalorhodopsin, Jaws, which is a chloride pump from *Haloarcula salinarum*. Jaws is a highly light-sensitive opsin with photocurrents three times as large as those of other known inhibitory opsins. This molecular tool is particularly useful for noninvasive neuronal inhibition in deep brain structures, or more effective inhibition in larger primate brains.

Despite the improvements in these inhibitory pumps, there are intrinsic limitations in using light-sensitive pumps to drive neuronal silencing. First, these pumps move only one ion per absorbed photon, which makes them inefficient compared to some of the currently available excitatory channel opsins that allow flow of ions through an open pore. Second, engineering approaches aimed to increase light sensitivity and enhanced long-term photocurrent stability (similar to SFOs) cannot be applied to pumps efficiently, as they depend on pore size. Two excellent recent studies used the crystal structure of channelrhodopsin hybrid C1C2, determined by Kato et al. (2012), to create a class of light-activated inhibitory chloride channels (Berndt et al., 2014; Wietek et al., 2014). iC1C2 allows for blue-shifted inhibition with fast kinetics, while SwiChR provides step function inhibition. This class of channels enables creation of different variants with enhanced light sensitivity, step function kinetics, and other characteristics similar to channelrhodopsin variants.

### Optogenetic Control of Intracellular Signaling

Vertebrate rhodopsins, unlike microbial opsins, are G protein–coupled receptors and act by modifying the activity of intracellular signaling pathways upon light activation. Utilizing this feature, Airan et al. (2009) developed a family of opsin-receptor chimeras called optoXRs. By replacing the intracellular loops of vertebrate rhodopsins with those of adrenergic receptors, they created light-sensitive proteins that could selectively recruit different signaling pathways upon light illumination in a targetable and temporally precise manner. Various other methods of optical control of biochemical activity are being developed, and have been reviewed (Tischer and Weiner, 2014).

### How Are Neurons Engineered to Express Opsins?

Efficient delivery and expression of opsin genes is critical for achieving spatiotemporally-resolved cell type–specific manipulation. This can be achieved in multiple ways. One method, popular because it allows for tight control over spatial localization of opsin expression, is through the use of viral vector targeting systems. Using this approach, an engineered virus containing an opsin gene driven by a specific promoter is injected into the brain region of interest. This method offers fast and robust expression. Various viral vectors such as lentivirus, adeno-associated virus, rabies virus, canine adenovirus, and herpes simplex virus—useful for different applications—have been used to introduce opsins to different systems, including mouse, rat, zebrafish, and primate models (Zhu et al., 2009; Zhang et al., 2010). However, the payloads (the length of genetic material a virus can carry) of these viruses are limited, thus limiting the size of the promoter and thereby reducing the diversity of cell types that can be specifically targeted with sufficient expression. This method is routinely used to target either neurons or excitatory neurons using the human synapsin (hSyn) or CaMKIIα promoters, and can be particularly effective when used for projection targeting. Using this approach, a fiber optic is implanted over a downstream brain region rather than over the infected cell bodies, which allows for pathway-specific modulation of neural activity (Yizhar et al., 2011a; Tye and Deisseroth, 2012). Some viral vectors have been developed to target specific genetically defined cell classes, but the majority of cell types require more genetic material to confer specificity than allowed by this approach.

This limitation can be avoided with the use of transgenic or knock-in animals that express an opsin in a particular neural population. Here, larger promoter fragments can be used to enable more specific expression. For example, a mouse line expressing opsin in neocortical layer 5 projection neurons was made using the Thy1 promoter (Arenkiel et al., 2007; Zhao et al., 2008), and lines have been created that express ChR2 in various specific cell types (Zhao et al., 2011). However, it takes more effort and time to generate a stable transgenic line, and the use of these animals is restricted because the spatial localization achieved through viral vector injection is lost. In addition, a new mouse line must be generated each time a new opsin is desired.

Cre recombinase-based mouse lines in combination with viral vectors, currently one of the dominant strategies used to target genetically defined cell types, offer more flexibility. Cre recombinase is an enzyme that catalyzes recombination between two loxP sites (specific 34 base pair sequences) that flank a gene or other genetic material of interest. Depending on the orientation of these sites, the genetic material lying between them will either be reversed or excised when the Cre enzyme is present, but unaffected when it is not. Typically, a viral vector containing an inverted and “floxed” (flanked by loxP) gene is delivered locally to a brain region of interest in a transgenic or knock-in mouse expressing Cre recombinase in a specific cell type. Although the virus will infect all cells, the opsin will only be flipped to the correct orientation and be functional in one cell type, since Cre is only present in these cells. Using this method, exquisitely tuned genetic and spatial (including projection-specific) targeting can be achieved. This method also has the advantage that it is “upgradable”: an existing Cre mouse line can be used for many different types of experiments with many different opsins with only a change of viral vector, an important consideration given the current speed of opsin development. This method has recently been extended by using alternative recombinases (e.g. Dre or Flp) in combination with Cre recombinase to allow targeting of cells defined by more than one protein marker (Fenno et al., 2014).

### How Are Neurons Illuminated?

#### Laser light sources

Lasers are widely used in optogenetics both because they permit the application of narrow bandwidth light (facilitating multimodal optical control with more than one opsin) and because they can be efficiently coupled to optical fibers. This last characteristic is a particular advantage in deeper brain structure manipulation with an implanted fiber optic. Diode-pumped solid state lasers with a maximum power of 100 mW are an appropriate choice in optogenetics (Adamantidis et al., 2007; Aravanis et al., 2007). The low divergence and high power of the laser beam enables it to be steered by multiple optical components, and it can be combined with other techniques to manipulate the activity of single neurons (Prakash et al., 2012) or achieve patterned stimulation (Packer et al., 2013).

Optical fibers can be used to deliver light to specific intracranial locations and permit optical control of deep brain structures. Small diameter optical fibers (~200 μm) minimize tissue damage and can be coupled efficiently to laser light sources (Warden et al., 2014). The fiber can be cut to the appropriate length to target a specific brain region and can either be fixed directly to the skull or inserted through a cannula to facilitate simultaneous pharmacological manipulations. Fibers may be placed bilaterally or used in other configurations to target large brain regions (Warden et al., 2014).

#### Light-Emitting Diode Light Sources

Light-emitting diodes (LEDs) are an attractive option for optical stimulation because of their low cost and narrow spectral tuning with diverse color options (Warden et al., 2014). However, the low efficiency of LED-optical fiber coupling limits the utility of LED light sources for some wavelengths of light due to low resultant power from the fiber tip, and heat generation may also prove problematic. However, their small size and low power requirements make LED light sources very useful for multisite illumination and portable wireless optogenetic devices (Wentz et al., 2011; Stark et al., 2012).

### What Are Some Pitfalls in Applying Optogenetics?

Optogenetics is a powerful tool that gives us the ability to dissect the function of neural circuits with an unprecedented level of precision, but there are some caveats to consider when planning an optogenetics experiment. First, optogenetic actuator expression and light delivery will generally not be uniform throughout the targeted population of neurons, likely resulting in different levels of activity in different cells. Second, optogenetic stimulation might push neurons outside of their normal physiological range, which may have important consequences. For example, neural silencers can induce non-physiological hyperpolarization, which can cause rebound excitation upon release of inhibition (Kravitz and Bonci, 2013; Hausser, 2014). Third, optogenetic stimulation synchronizes the activity of all the cells within the targeted population, unless a reagent such as a step-function opsin is chosen. Such synchronous activation is not physiological, as the differences in individual neuronal firing patterns within a target population such as spike rate and phase (with respect to the local field potential) are lost.

Fourth, optogenetic tools indiscriminately drive all cells within a genetically defined targeted population and cannot modulate subsets of this population. This problem is being addressed by several methods. INTERSECT (intron recombinase sites enabling combinatorial targeting) utilizes multiple recombinases (for example Cre, Dre, and Flp), each expressed in a different cell type, to target cell populations defined by more than one marker (Fenno et al., 2014). Other methods use optogenetic actuators driven by immediate early genes to target only recently active cells (Liu et al., 2012; Ramirez et al., 2013) as an alternative method for increasing specificity.

Fifth, direct illumination and stimulation of axonal membranes can cause antidromic activation of both neuronal cell bodies and collaterals to other brain regions, resulting in activation of different circuits and reducing the specificity of manipulation, although pharmacological approaches can be used to demonstrate synapse specificity if this is a concern (Tye et al., 2011). This potential problem is not a concern when inhibitory opsins are used. Finally, expression of opsin proteins has the potential to alter the function of intrinsic cellular machinery, and physiological differences may result from the insertion of large numbers of foreign ion channels or pumps into the cellular membrane. Thus, care should be taken when applying optogenetic tools.

## Conclusion

Optogenetics has changed the landscape of neuroscience, and has enabled a new generation of experiments that dissect the causal roles of specific neural circuit components in normal and dysfunctional behavior. It has been used to increase our understanding of the neural circuits underlying mood disorders (Covington et al., 2010; Tye et al., 2011, 2013; Lobo et al., 2012; Warden et al., 2012; Kim et al., 2013; Lammel et al., 2014; Sidor et al., 2015), addiction (Lobo et al., 2010; Chen et al., 2013), Parkinson’s disease (Gradinaru et al., 2009; Kravitz et al., 2010), obsessive compulsive disorder (Ahmari et al., 2013; Burguière et al., 2013), social behavior (Yizhar et al., 2011b; Yizhar, 2012; Dölen et al., 2013; Gunaydin et al., 2014), and reward (Tsai et al., 2009; Witten et al., 2010, 2011; Stuber et al., 2011a, 2011b; Van Zessen et al., 2012), among others (Deisseroth, 2014). The past decade has seen an explosion in the development of new optogenetic tools, both through discovery and engineering, and there is now a toolbox of exquisitely tuned opsins that can be used to stimulate and inhibit neural activity and control intracellular signaling cascades (Mattis et al., 2012; Tye and Deisseroth, 2012). Development of optogenetic tools continues, and some of the most exciting current research is directed at the refinement of combinatorial optogenetic approaches and integration of optogenetic control with imaging of genetically defined neural populations (Akerboom et al., 2013; Fenno et al., 2014; Rickgauer et al., 2014; Warden et al., 2014). The coming years should see exciting progress in the development and application of these tools to deconstruct the neural circuits underlying normal behavior and their dysfunction in psychiatric disease.

## Statement of Interest

The authors have no conflicts of interest to declare.
